# Dentists' Awareness of Physical Therapy in the Treatment of Temporomandibular Disorders: A Preliminary Study

**DOI:** 10.1155/2018/1563716

**Published:** 2018-02-28

**Authors:** Inae C. Gadotti, Corey Hulse, Julia Vlassov, Derek Sanders, Daniela A. Biasotto-Gonzalez

**Affiliations:** ^1^Department of Physical Therapy, Florida International University, Miami, FL, USA; ^2^Orthodontics Only Practice, Miami, FL, USA; ^3^Rehabilitation Sciences Program, University of Nove de Julho, São Paulo, Brazil

## Abstract

**Background:**

Physical therapy (PT) has been shown to be one of the most effective conservative treatments for temporomandibular disorders (TMD). Not all dentists are aware of the importance of the collaboration with physical therapists in the treatment of TMD pain.

**Objectives:**

To determine the awareness of dentists in Florida about the importance of PT for TMD pain and to create awareness related to collaborations.

**Methods:**

An online questionnaire was used. A contact list of dentists was obtained from the Florida Dental Association. The overall awareness and information on patient referral were presented per dentist specialty.

**Results:**

A total of 256 dentists completed the survey. Prior to the survey, 41% of the dentists reported not aware that PTs can treat TMD patients. Oral surgeons and orthodontists were more aware about PT compared to other specialties. After the survey, 81% of the dentists were more likely to refer their TMD patients to PT, and 80% were interested to know more about the benefits of collaborations.

**Conclusion:**

This study shows the lack of dentists' awareness in Florida about the benefits of PT for TMD treatment. This study increased the awareness of the surveyed dentists in Florida about the benefit from a multidisciplinary approach.

## 1. Introduction

The temporomandibular joint (TMJ) is part of the musculoskeletal system responsible for mandibular function which includes mastication, phonation, and deglutition [1]. Temporomandibular disorders (TMD) are defined as a musculoskeletal disorder affecting the TMJ, the masticatory muscles, and associated structures including dental occlusion and the cervical spine [2, 3]. TMD are the most common chronic orofacial pain condition, and it can significantly affect the patient's quality of life by diminishing the individual's ability to work and interact in social environment [3].

Approximately 10% of the population has pain in the TMJ [4], and 3.6%–7% of the population will seek treatment due to the severity of their symptoms [3, 5]. TMD signs and symptoms may include local pain in the TMJ and/or masticatory muscles, limited mouth movements, TMJ sounds, and headaches [5–7]. Cervical spine disorders were shown to be associated with TMD pain 70% of the time [7–11].

The different types of TMD are classified based on the Diagnostic Criteria for Temporomandibular Disorders (DC/TMD) [12]. TMD can be acute or chronic, simple or complex with persistent and associated cognitive, psychosocial, and behavioral factors [12]. A multidisciplinary approach is particularly important for successful treatment of chronic TMD cases [13]. Treatment of TMD pain may involve dentists, physical therapists (PTs), speech pathologists, physicians, and psychologists. An ideal treatment option would be the one that is least invasive and most cost-effective, while considering the TMD associated factors such as parafunctional habits, poor posture, widespread pain, poor sleep, and depression [3].

Physical therapy (PT) is one of the most effective conservative treatments for TMD pain [14]. PT is among other noninvasive therapies including behavioral therapy and occlusal appliances that were shown to improve patients with TMD [15]. The most important contribution by PTs is the identification of the musculoskeletal components that contribute to the symptoms of the patient [7]. Because the TMJs are part of the musculoskeletal system, PTs can treat TMJ-related pain with similar interventions as they would in most other body joints. PT includes a large number of modalities to treat TMD pain secondary to inflammation, masticatory muscle pain, TMJ hypo/hypermobility, disc displacement, bruxism, and fibrous adhesion [7]. Based on systematic reviews [16, 17], manual therapy, jaw exercises, and postural reeducation were shown to be effective to reduce pain and improve mobility/function in TMD patients.

More collaboration between dentists and PTs for the management of TMD pain is needed so as to improve the treatment outcomes of these patients. Not all dentists are aware of the importance of involving PTs in the treatment of TMD pain. The awareness of dentists from Florida about PT's role for TMD treatment is unknown. Therefore, the primary objective of this research was to determine the current level of awareness of dentists in Florida about the importance of PT and the collaboration with PTs in the treatment of TMD. The secondary objective was to increase the awareness level of dentists regarding the importance of PT and the benefits of the collaboration between dentists and PTs in TMD treatment to potentially increase collaborations between dentists and PTs in the treatment of TMD for best outcomes.

## 2. Materials and Methods

### 2.1. Study Design

This was a cross-sectional descriptive study approved by the Institutional Research Board from Florida International University (IRB-14-0205).

### 2.2. Participants

Dentists in Florida with an active dental license and members of the Florida Dental Association were contacted. A contact list of dentists was obtained from the Florida Dental Association. The dentists were contacted by an email, which included a statement of the study objectives and a link to the online survey. By completing and submitting the survey, the dentists were informed that they were consenting to participate in the study. The dentists were informed that no identifiable information will be published or released and that participation is voluntary. All data were confidentially analyzed. In addition, they were informed that they will receive no compensation for participating in the study. However, an educational brochure with information related to PT for treating TMD pain was available to them upon completion of the survey. A reminder email was sent 3 times every 2 weeks from the initial recruitment email.

### 2.3. Questionnaire

A questionnaire was created using Qualtrics online survey software (Qualtrics Labs Inc., Provo, Utah). The survey was revised by 2 dentists to gather feedback for improvements. Feedback was considered and changes to the survey were implemented. The questionnaire included a total of 24 questions: 7 related to demographics, 12 on TMD patient population and referrals, and 5 related to general knowledge (Appendix 1). The online survey was estimated to take approximately 5–10 minutes to complete.

### 2.4. Data Analysis

Descriptive statistics were calculated to analyze the responses. Data were presented as total number of participants (*n*) and frequency (%). Written information provided by some dentists was considered and presented. The overall knowledge related to PT among the respondents and information on patient referral were calculated and presented per dentist specialty.

## 3. Results

### 3.1. Participants' Demographics and Characteristics

From over 10,000 emails sent, a total of 256 dentists completed the survey (response rate of 2.5%). The mean age of the participants was 51 years with a range of 26 to 78 years, and 172 of the participants (67%) were male. Ninety-seven percent of the participants (243) had earned their professional doctoral degree, 2% [5] had earned an academic master's degree, and 0.4% [1] had earned an academic doctoral degree (PhD). Two hundred twelve participants (86%) reported practicing dentistry in a private practice setting, and most of the participants (41%) practiced for 21 to 35 years. Twenty-eight percent (65) were from South Florida District followed by West Cost District (24%) and Central Florida District (19%). The majority of the participants (73%) were general dentists followed by orthodontists (8%) and other specialties (18%), which included pediatric dentistry, TMJ and orofacial pain specialist, and neuromuscular dentistry.  Table 1 shows detailed demographic and characteristics of participants.

Thirty-nine percent of the dentists (95) had never taken continuum education course on TMD. For those dentists reporting yes for taken courses on TMD, the courses included topics related to etiology and treatment of TMJ disorders; occlusion; bite plane therapy; TMD and occlusion; splint therapy, medication and restorative therapy; surgical and nonsurgical treatment; facial pain; myofascial pain and TMD; arthroscopic surgery, traumatic derangement; joint prosthetics and replacement; and occlusion and posture. Six dentists reported PT as part of the topic in the continuum education course taken.

### 3.2. TMD Patients' Information

More than half of the dentists surveyed (57%) estimated anywhere from 1 to 15% of their patients suffered from TMD symptoms. Only 2 dentists reported not having seen these type of patients, and 17 dentists (7%) reported that more than 55% of their patients have TMD. The most common characteristics of TMD evaluated and/or treated were parafunction habits (89%), muscle tightness/tender points (75%), occlusion alterations (75%), and headaches (69%). The least common characteristics were TMJ hypermobility (26%) and TMJ degeneration (38%). Other TMD characteristics evaluated and/or treated included condyle fracture, traumatic injury, neuropathic pain, and craniocervical issues. Only 7 dentists (3%) reported never evaluating a patient with a TMJ-related problem. Methods of TMD evaluation most often used by dentists included observing jaw movements during opening/closing (86%), evaluating for dental occlusion (84%), TMJ palpation (83%), and signs of parafunctional habits (81%). Other methods reported to evaluate TMD patients included neck range of motion, radiographs, photographs, MRI, diagnostic anesthesia, biopsychosocial measurements, and surface electromyography (sEMG). Most of the patients (55%) presented a chronic condition during the initial evaluation, as opposed to the acute (25%) and subacute conditions (20%).

When asking the dentists whether or not their TMD patients also presented with neck pain, poor posture, and/or cervicogenic headache, 13%, 34%, and 32% reported never evaluating these conditions, respectively. From those who had evaluated, 76%, 58%, and 59% reported finding these conditions, respectively, present in their patients.

### 3.3. Treatment and Referral

**Figure 1:**
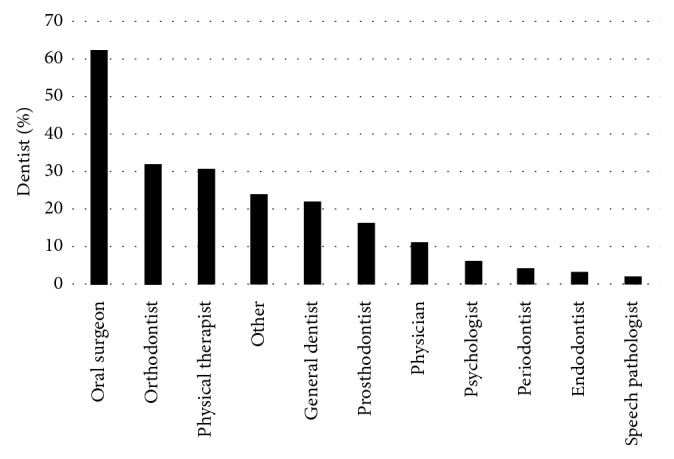
Health care providers TMD patients are referred to.

The most frequent methods used to treat TMD patients (if patients are not referred) were the use of bite splints (90%), prescription medication (62%), followed by occlusion correction (58%). However, 69 dentists (30%) utilized other treatment methods including ice/heat, arthrocentesis, diet alteration, jaw and neck exercises, botox, trigger point injection, thermotherapy and cryotherapy, and soft tissue massage. Eighty-six percent (86%) reported referring TMD patients to other health care providers. Most of these dentists (70%) reported referring up to 25%. Thirteen percent (13%) reported referring 75–100% of their TMD patients. The health care providers to which TMD patients were most commonly referred were oral surgeons (62%), orthodontists (32%), and PTs (31%) (Figure 1). Other providers described included TMJ/orofacial pain specialist, chiropractor, massage therapist, gnathologist, neuromuscular dentist, endocrinologist, neurologist, osteopath, and ENT.  Table 2 shows the distribution of TMD patient referral per dentist specialty. The specialty that refers the most the TMD patients to PTs was oral surgeons (80% of them) followed by orthodontists (55%).

**Figure 2:**
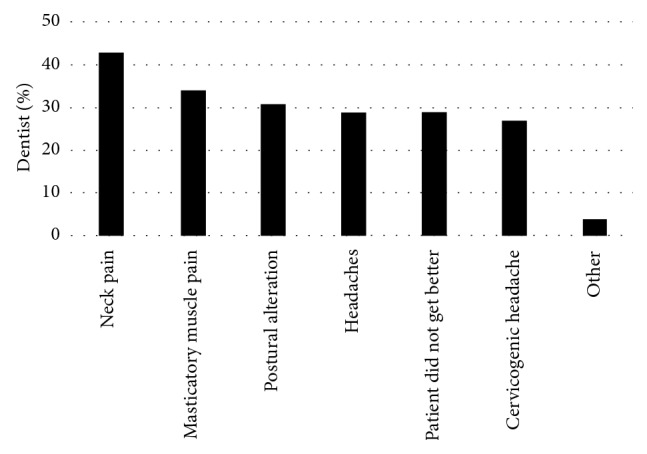
Conditions under which TMD patients are referred to physical therapy.

The most common reasons for TMD patient referral to a PT included neck pain (43%), masticatory muscle tenderness (34%), and postural alterations (31%) (Figure 2). The most common reason for not referring a patient to PT was that they did not know about the benefits of PT to the patient (58% of them). Other reasons reported were “lack of knowledge of a PT that treats TMJ or contact information,” “insurance payment,” “no formal referral system set in place,” and the belief that “PT is only a temporary fix” or “it is out of their skill set.” In fact, 41% of all the dentists surveyed had no knowledge that PTs were capable of treating patients with TMD.

### 3.4. Physical Therapy Awareness

Prior to the survey, 41% of the dentists reported not aware that PTs can treat patients with TMD by, for example, reeducating jaw movements, and restoring masticatory muscles (Table 3). In addition, 32% of the dentists reported not aware that cervical spine may be involved with masticatory region pain. Oral surgeons and orthodontists were more aware about PT for TMD management compared to other specialties (Table 3).

After asking the dentists surveyed if they are more likely to refer any of their TMD patients to a PT after participating in the survey, 184 dentists (81%) are more likely to refer to a PT by answering yes or may be. Reasons for not being likely to refer or may be are as follows: “do not know how the process is to refer it,” “none have offered services,” “too specific of treatment for PT to be helpful,” “insurance issues,” “not allowed to refer,” “PT treatment only helps temporarily,” or “do not know where to refer in my area.” At the end of the survey, 80% of the dentists (180) were interested to know more about the benefits of the collaborations with PTs to treat TMD patients. The proportion of dentists' interest on knowing more about the benefits of the collaborations with PTs to treat TMD patients is also shown in Table 4 by dentist specialty.

## 4. Discussion

This was the first study evaluating the awareness of dentists in Florida about the importance of the multidisciplinary approach with PT for the management of TMD pain using an online questionnaire. Information about TMD patients treated by dentists, TMD patient referral, and their interest to know more about PT for the management of TMD pain is described. However, the results of this study should be interpreted with caution because the response rate was very low, and therefore the generalizability of the findings is questionable. However, this preliminary study presents relevant information regarding the current level of awareness among the participants and can help further increase the level of collaboration between PTs and dentists in the treatment of TMD. From the 88 of surveyed dentists who had never referred a TMD patient to a PT, 65 of them (74%) were not aware of the benefits of PT in treating TMD patients. Perhaps, the referral to PTs would be greater if more dentists were aware of PT for TMD patients. In fact, after this survey, 81% of the dentists are more likely to refer a TMD patient to PT. Other studies should investigate if the referral is actually happening in Florida. According to a dentist author in his article with a PT colleague [2], 50% of all his patients are referred to PT. Based on the TMD patients' characteristics reported by the dentists in our survey, it appears that most of them could be referred to PT for further treatment.

Approximately 1/3 of dentists surveyed do not evaluate their TMD patients for poor head and neck posture and the presence of cervicogenic headaches. Also, 13% of dentists do not evaluate for the presence of neck pain. As previous researches show correlation between TMD pain and the presence of cervical spine disorders including neck pain and poor posture [7, 9–11], dentists should be aware of these disorders in their patients in order to possibly refer the patients to PTs for further treatment and collaboration. On the other hand, PTs should be also aware of any possible tooth-related pain or dental occlusion problems related to TMD during their evaluation in order to possibly refer the patient to the dentist. For example, if parafunction habits are common in patients with TMD, a dental splint may be fabricated by the dentist. At the same time, PTs can deprogram the masticatory muscles with soft tissue massage and intraoral mobilizations before the exercises. TMD patients will have better treatment outcomes if both dentists and PTs work together [2]. In a randomized control trial, patients who received a combination of dental splint therapy with PT had greater gains in mouth range of motion than splint therapy alone [18].

The health care providers to which TMD patients were most commonly referred to were oral surgeons (62%). Oral surgeons were the health care providers who refer the most the TMD patients to PTs. Table 4 shows that almost 70% of general dentists refer patients to oral surgeons. It seems that TMD patients are mostly referred to PT for postsurgery treatment. Studies indicate that PT has a positive effect in relieving pain and restoring TMJ function after surgery [19, 20]. However, if applicable, TMD patients should be referred to PT before a nonconservative treatment such as surgery is considered. In addition, in the cases where surgery is needed, PT should be considered as a presurgical treatment in order to prepare the patients for surgery. The benefits of the surgery may be increased if PT is done before surgery. Gladly, 55% of the orthodonticts refer TMD patients to PT.

A lack of dentists' awareness about the benefits of PT for the treatment of TMD patients leads to less patient referral and collaborations with PT. In fact, the most common reason for not referring a patient to a PT was the lack of awareness of PT benefits (58%). More awareness related to the relationship between cervical spine and orofacial symptoms is needed as 32% of the dentists were not aware. For instance, cervical spine postural reeducation is recommended for TMD patients in addition to manual therapy and jaw exercises [16]. PT is considered an integral part of TMD treatments [13]. Physical therapy, as well as behavioral therapy and occlusal appliances help to improve patients with TMD [15]. Therefore, according to the authors of this study, information on the role of PT on TMD treatment should be part of seminars and lectures in the curriculum of Dentistry Programs to inform them on the importance of interdisciplinary treatment of TMD patients.

From the dentists' awareness about the benefits of PT for the management of TMD patients prior to the survey (146 dentists), 62 (43%) are more likely to refer patients to PTs after participating in the survey. But 58% of them said may be or are not likely to refer (31% and 27%, resp.). Therefore, the fact that some dentists are aware about the benefits of PT does not mean that the referral is happening. One of the possible reasons for the low rate of referral of patients with TMD to PTs is the lack of available PTs with expertise in treating TMD because not all PTs are trained and confident about providing care to TMD patients. The number of PTs with specialized training and advanced education in the area of TMD such as PTs certified by the Physical Therapy Board of Craniofacial and Cervical Therapeutics (many of them members of American Academy of Orofacial Pain) represents a small fraction of the American Physical Therapy Association (APTA). Therefore, more education related to TMJ, TMD, and the multidisciplinary approach between dentists and PTs in the pain management of TMD patients should be also reinforced in all PT Programs. Interestingly, one comment received by a dentist was that there is a need to also educate the PTs regarding collaborations. A study with PTs about their knowledge to treat TMD patients should be conducted. Their capabilities to collaborate with dentists should be also measured. If more PTs are capable of treating these patients the likelihood of dentists to refer their patients may increase. Information related to should be part of the curriculum of all PT programs.

TMD can be complex because patients may present different conditions including arthralgia, myalgia, myofascial pain, disc displacement disorders, degenerative joint disease, and headache attributed to TMD among other classifications [12]. In addition, other associated factors may be present such as generalized pain, sleep disturbances, and depression. Therefore, diagnosis and treatment of these patients is challenging [12], and a multidisciplinary team is recommended to treat TMD. However, not all disciplines are necessarily needed for treating all cases of TMD. Patients' symptoms should be considered to decide which professionals need to be involved.

### 4.1. Study Limitations

From over 10,000 contacted dentists, only 2.4% of them responded to the survey. The authors believe that not all email addresses were updated in the list provided and that could have affected the amount of responses received. In order to maximize participation, the survey was made to be short (5–10 minutes to complete). In addition, a brochure with information about PT treatment was available upon participation. Other strategies to increase participation should be considered in future studies.

This study included dentists from the State of Florida only. Future studies should include larger sample (higher response rate) by not only including dentists from other states, but also including more dentists from the other specialties. The majority of the dentists who responded the survey were general dentists (73%). Results may not be generalizable when data were analyzed by specialty. Therefore, the results should be interpreted with caution; future studies with higher response rates and including different dental specialties are needed.

## 5. Conclusion

According to this survey, a large percentage of the dentists that completed the survey were not aware of the benefits of PT in treating TMD pain. This study helped to increase the awareness level of the surveyed dentists in Florida about the importance of physical therapy and the benefit from the multidisciplinary approach with PT to their patients. Most of the dentists surveyed (80%) were interested to know more about the benefits of the collaborations with PTs to treat TMD patients. This is important as the increased awareness of dentists about the importance of physical therapy and the interest to know more about the benefits may increase collaborations between dentists and PTs in the treatment of TMD patients in Florida. TMD patients are more likely to be benefited from those collaborations. Future studies should investigate if collaborations between dentists and PTs are increasing and if TMD patients treatment benefit from those collaborations.

## Figures and Tables

**Table 1:** Participants' demographics and characteristics.

Variable	Value
Age, years (mean, standard deviation, and range)	51 ± 13 (26–78)
Gender, male/female (total number, percentage)	172 (67%)/84 (33%)
Highest level of education (total number, percentage)	
Professional doctorate	243 (97.5%)
Academic master	5 (2%)
PhD	1 (0.4%)
Areas of practice (total number, percentage)	
General dentist	178 (73%)
Orthodontists	20 (8%)
Endodontist	9 (4%)
Prosthodontics	5 (2%)
Periodontist	7 (3%)
Oral surgeon	7 (3%)
Other	18 (7%)
Years of practice (total number, percentage)	
0–5	36 (15%)
6–10	26 (11%)
11–15	21 (9%)
16–20	20 (8%)
21–25	32 (13%)
26–30	32 (13%)
31–35	37 (15%)
36–40	23 (9%)
41–45	13 (5%)
46–50	5 (2%)
51–55	1 (0.4%)
>56	1 (0.4%)
Continuum educational course in TMD	
Yes	150 (61%)
No	95 (39%)

**Table 2:** Health care providers TMD patients are referred to by dentist specialty (total number and percentage).

	Dentist specialty						
Patients referral to	General dentist	Orthodontist	Endodontist	Prosthodontist	Periodontist	Oral surgeon	Other
General dentist	26	9	4	0	2	1	3
	17.81%	45.00%	44.44%	—	40.00%	20.00%	27.27%
Orthodontist	53	0	2	0	1	4	4
	36.30%	—	22.22%	—	20.00%	**80.00%**	36.36%
Endodontist	3	1	0	0	1	0	0
	2.05%	5.00%	—	—	20.00%	—	—
Prosthodontist	18	2	4	2	2	2	1
	12.33%	10.00%	44.44%	50.00%	40.00%	40.00%	9.09%
Periodontist	6	0	2	0	0	0	0
	4.11%	—	22.22%	—	—	—	—
Oral surgeon	100	10	3	1	3	0	7
	**68.49%**	50.00%	33.33%	25.00%	**60.00%**	—	63.64%
Physical therapist	42	11	1	1	2	4	1
	28.77%	**55.00%**	11.11%	25.00%	40.00%	**80.00%**	9.09%
Physician	15	5	0	1	0	1	0
	10.27%	25.00%	—	25.00%	—	20.00%	—
Psychologist	6	5	0	1	0	0	0
	4.11%	25.00%	—	25.00%	—	—	—
Speech pathologist	1	1	0	0	0	0	1
	0.68%	5.00%	—	—	—	—	9.09%
Other	34	6	3	1	1	0	4
	23.29%	30.00%	33.33%	25.00%	20.00%	—	36.36%

**Table 3:** Dentists' awareness about physical therapy treatment for TMD by dentist specialty.

	Aware	Not aware
General dentist	96 (58%)	70 (42%)
Orthodontist	14 (70%)	6 (30%)
Endodontist	5 (56%)	4 (44%)
Prosthodontist	2 (40%)	3 (60%)
Periodontist	4 (57%)	3 (43%)
Oral surgeon	6 (100%)	0 (0%)
Other	8 (50%)	8 (50%)
Total	135 (59%)	94 (41%)

**Table 4:** Dentists' interest on the benefits of collaborations with physical therapists to treat TMD patients by dentist specialty.

	Yes	No
General dentist	133 (83%)	28 (17%)
Orthodontist	16 (80%)	4 (20%)
Endodontist	6 (67%)	3 (33%)
Prosthodontist	4 (80%)	1 (20%)
Periodontist	4 (57%)	3 (43%)
Oral surgeon	5 (83%)	1 (17%)
Other	11 (69%)	5 (31%)
Total	179 (80%)	45 (20%)

## Appendix

### Survey

Questions related to demographic and work experience:

Do you agree to participate?

Yes, I consent to participate.

1. Gender
  - Male
  - Female
2. How old are you? Please enter in years._____________________________
3. Highest level of Dentist degree completed
  - Bachelor's degree
  - Master's degree
  - Doctoral degree
4. Do you currently practice?
  - Yes
  - No
5. How many years have you been practicing?
  - 0–5
  - 6–10
  - 11–15
  - 16–20
  - 21–25
  - 26–30
  - 31–35
  - 36–40
  - 41–45
  - 46–50
  - 51–55
  - More than 55 years
6. What is your Florida Association District in which you currently work?
  - Atlantic Coast District
  - Central Florida District
  - Northeast District
  - South Florida District
  - Northwest Florida District
  - West Coast District
7. Which title best describes you?
  - General Dentist
  - Orthodontist
  - Endodontist
  - Prosthodontist
  - Periodontist
  - Oral Surgeon
  - Other: ________________________________
8. Have you ever taken continuing education courses on temporomandibular disorders (TMD)?
  - Yes
  - No
  - If yes, what topic(s) specifically?______________________________________ ______________________________________ ______

Questions related to TMD patients population and referrals

(9) What percentage of your patients would you estimate to suffer from TMD symptoms (temporomandibular joint-TMJ or muscle pain, clicking, popping, headaches, etc.)?
  - 0%
  - 1–5%
  - 5–15%
  - 15–25%
  - 25–35%
  - 35–45%
  - 45–55%
  - 55–75%
  - 75–90%
  - 90%–100%
(10) What type of TMD you have had evaluated and/or treated? (you may select more than one)
  - TMJ disc displacement
  - TMJ degeneration
  - TMJ hypermobility
  - TMJ hypomobility/mouth opening limitation
  - Muscle tightness/tender points
  - Occlusion alterations
  - Parafunction habits (i.e., bruxism)
  - Headaches
  - Never evaluated or treated a patient with TMJ-related problem (If true, skip to question 15.)
  - Other: Please add:
(11) From the options below, what do you normally include in the evaluation of these patients? (You may select more than one)
  - TMJ palpation
  - Masticatory muscles palpation
  - Jaw movements during opening/close
  - TMJ sounds
  - Signs of parafunction habits
  - Dental occlusion
  - None of the above
  - Other:
(12) What stage of the disorder have most of your patients with TMD presented with during evaluation?
  - Acute
  - Subacute
  - Chronic
(13) Does any of the conditions below were present in your patients with TMD during evaluation?
            Yes No Never evaluated
  Neck pain
  Poor posture
  Cervicogenic headache
(14) When treating patients with TMD, what methods do you normally use? (you may select more than one)
  - Bite splints/occlusal guards
  - Occlusion correction/braces
  - Prescription of medication
  - If other treatment, please specify __________
(15) Do you refer patients with TMD to other practitioners?
  - Yes
  - No (If true, skip to question 19)
(16) What percentage of these patients do you refer?
  - 0–5%
  - 5%–25%
  - 25%–50%
  - 50%–75%
  - 75%–100%
(17) Which health care provider/specialty do you refer to specifically? (You may select more than one option).
  - General Dentist
  - Orthodontist
  - Endodontist
  - Prosthodontist
  - Periodontist
  - Oral Surgeon
  - Physical Therapist
  - Physician
  - Psychologist
  - Speech pathologist
  - If other, please specify ________________
(18) If you had referred a patient with TMD to a physical therapist, what would be the reason(s) for the referral (patient symptoms/condition)? (You may select more than one option).
  (a) Neck pain
  (b) Postural alteration (e.g., forward head posture)
  (c) Masticatory muscle tenderness
  (d) Headaches
  (e) Cervicogenic headache
  (f) Patient did not get better after your treatment
  (g) Other_______
    Never referred to physical therapy
(19) If you have never referred a patient with TMJ-related problem to a physical therapist, what is/are the reasons?
  - No need of PT treatment
  - Did not know about the benefit of physical therapy to the patient
  - Other:

Questions related to general knowledge

(20) Prior to this survey, were you aware that physical therapist can treat patients with TMD by, for example, reeducating jaw movements and restoring masticatory muscle function?
  - Yes
  - No
(21) Prior to this survey, were you aware that cervical spine pain may be involved as a cause of masticatory region pain?
  - Yes
  - No
(22) Prior to this survey, were you aware that the evidence suggests that physical therapy can improve TMD symptoms with oral exercises, manual therapy, and postural reeducation?
  - Yes
  - No
(23) After participating in this survey, are you more likely to refer a patient with TMD to a physical therapist when needed?
  (a) Yes
  (b) May be
  (c) No
  29.a. If no, why?_________
(24) Would you be interested in learning more about the benefits of the collaborations with physical therapists to treat TMD patients?
  - Yes
  - No
